# Two Alternative Splicing Variants of AtERF73/HRE1, HRE1α and HRE1β, Have Differential Transactivation Activities in *Arabidopsis*

**DOI:** 10.3390/ijms21196984

**Published:** 2020-09-23

**Authors:** Hye-Yeon Seok, Jimin Ha, Sun-Young Lee, Hyoungjoon Bae, Yong-Hwan Moon

**Affiliations:** 1 Institute of Systems Biology, Pusan National University, Busan 46241, Korea; seokhyeon@pusan.ac.kr; 2 Department of Integrated Biological Science, Pusan National University, Busan 46241, Korea; idef_@naver.com (J.H.); laptopdog@naver.com (H.B.); 3 Biological Systems and Engineering Division, Lawrence Berkeley National Laboratory, Berkeley, CA 94720, USA; symoonlee@lbl.gov; 4 Department of Molecular Biology, Pusan National University, Busan 46241, Korea

**Keywords:** alternative splicing, *Arabidopsis*, HRE1, hypoxia, root development, transactivation

## Abstract

AtERF73/HRE1 is an AP2/ERF transcription factor in *Arabidopsis* and has two distinct alternative splicing variants, HRE1α and HRE1β. In this study, we examined the differences between the molecular functions of HRE1α and HRE1β. We found that HRE1α and HRE1β are both involved in hypoxia response and root development and have transactivation activity. Two conserved motifs in the C-terminal region of HRE1α and HRE1β, EELL and LWSY-like, contributed to their transactivation activity, specifically the four E residues in the EELL motif and the MGLWS amino acid sequence at the end of the LWSY-like motif. The N-terminal region of HRE1β also showed transactivation activity, mediated by the VDDG motif, whereas that of HRE1α did not. The transactivation activity of HRE1β was stronger than that of HRE1α in *Arabidopsis* protoplasts. Both transcription factors transactivated downstream genes via the GCC box. RNA-sequencing analysis further supported that both HRE1α and HRE1β might regulate gene expression associated with the hypoxia stress response, although they may transactivate different subsets of genes in downstream pathways. Our results, together with previous studies, suggested that HRE1α and HRE1β differentially transactivate downstream genes in hypoxia response and root development in *Arabidopsis*.

## 1. Introduction

To survive under stressful conditions, plants change the expression patterns of stress-responsive genes. In this process, transcription factors are the *trans*-acting elements that play major roles in regulating gene expression by binding to *cis*-acting elements [1]. The APETALA2/ethylene-responsive factor (AP2/ERF) transcription factor family consists of transcription factors that are mainly plant-specific but are also found in protists, cyanobacteria, and phages [2]. Members of the AP2/ERF superfamily are encoded by 145 loci in *Arabidopsis* and 167 loci in rice [3,4,5]. AP2/ERF transcription factors are divided into four subfamilies: AP2, ERF, dehydration-responsive element-binding factor/C-repeat-binding factor (DREB/CBF), and RAV [4,6]. They function as either transcriptional activators or repressors in gene regulation. The specific conserved activation domains of these activators are not well-known, except for the LWSY motif, which has been identified at the C-terminus of ERFs and DREB/CBFs [7,8,9,10,11]. Acidic amino acid residues, such as aspartate (D) and glutamate (E), participate in the transactivation activity of these activators [12]. In contrast, repressors have distinct repressor domains, such as the ERF-associated amphiphilic repression motif, TLLLFR motif, and B3 repression domain [13,14,15]. Transcription factors in the ERF subfamily bind to the GCC box (5′-AGCCGCC-3′) and/or to dehydration-responsive element/C-repeat (DRE/CRT) (5′-A/GCCGAC-3′) to regulate the expression of downstream genes [16,17].

The splicing of pre-mRNA is a crucial step in the expression of the information encoded in eukaryotic genomes [18]. Alternative splicing occurs when splice sites are differentially recognized; more than one transcript and multiple potential proteins are generated from the same pre-mRNA [18]. The splice sites that are selected under particular cellular conditions are determined by the interactions of proteins, globally designated as splicing factors, which guide spliceosomal components and subsequently the spliceosome to the respective splice sites [19,20,21].

Abiotic stresses, such as heat, cold, salinity, and drought, markedly change alternative splicing patterns, thus implementing changes in gene expression as a part of the adaptive responses of plants to adverse environments. Splicing factors themselves also change their expression or activity under stress conditions [22]: distinctive splicing variants are expressed in different tissues or undergo degradation via nonsense-mediated decay [22]. Splicing variants may also vary with regard to their subcellular localization or have different biological functions; for example, *HfsA2* generates a truncated protein by alternative splicing under heat stress and acts as a positive regulator of its own transcription [23]; *bZIP60*, encoding a key transcription factor in the unfolded protein response, generates a nucleus-targeted alternative splicing variant under heat stress, while it generates an endoplasmic reticulum-localized protein under normal conditions. The splicing variant of bZIP60 localized in the nucleus activates the transcription of genes regulating protein folding and degradation [24,25]. In contrast, *ZIFL1* generates two alternative splicing variants encoding full-length and truncated proteins localized in the tonoplast of root cells and the plasma membrane of leaf stomatal guard cells, respectively; the full-length protein regulates auxin transport in the root cells, while the truncated protein mediates drought tolerance in the stomatal guard cells [26]. Although the regulation and functions of abiotic stress alternative splicing variants have been studied in plants, their role and downstream pathways in hypoxia responses have not yet been investigated, except in a small number of high-throughput studies [27,28]. Hypoxia is a growth-limiting factor, especially for non-oxygen-evolving organs or green tissues under dark conditions [29,30,31], leading to a decrease in ATP production and a subsequent energy crisis affecting numerous plant processes. To circumvent a negative energy status, plants have developed several strategies, including a switch to anaerobic fermentation to regenerate the NAD^+^ required to sustain glycolytic ATP production [29,30].

AtERF73/HRE1 (HRE1) is an AP2/ERF transcription factor in *Arabidopsis* that belongs to group VII of the ERF subfamily and contains one AP2/ERF domain [32]. *HRE1* has two distinct alternative splicing variants, *HRE1α* and *HRE1β* [33], whose overexpression confers tolerance to flooding or anoxia [33,34]. Moreover, *HRE1α*-overexpressing transgenic plants (OXs) exhibit increased primary root elongation due to elevated root cell division [33]. It has been also reported that HRE1α has transactivation activity, supposedly imputable to its C-terminal region [33]. Although previous studies have examined each splicing variant, no study has yet compared HRE1α and HRE1β, nor their transactivation activity, the domains responsible for their activity, and the downstream genes that they affect.

In this study, we assessed the functional differences between HRE1α and HRE1β. Expression pattern analyses suggested that *HRE1β* plays a more important role than *HRE1α* in the hypoxia response, and analysis of *HRE1α* OXs and *HRE1β* OXs showed that both splicing variants are involved in the hypoxia response and root development. Our results demonstrated that both HRE1α and HRE1β have two motifs in their C-terminal region that contribute to their transactivation activity. HRE1β has an additional motif in its N-terminal region that is also involved in its transactivation activity. RNA-sequencing (RNA-Seq) analysis of genes downstream of *HRE1α* and *HRE1β* revealed that both these splicing variants might function differentially as transcriptional activators via the GCC box in *Arabidopsis*.

## 2. Results

### 2.1. Structural Comparison of HRE1α and HRE1β

**Figure 1:**
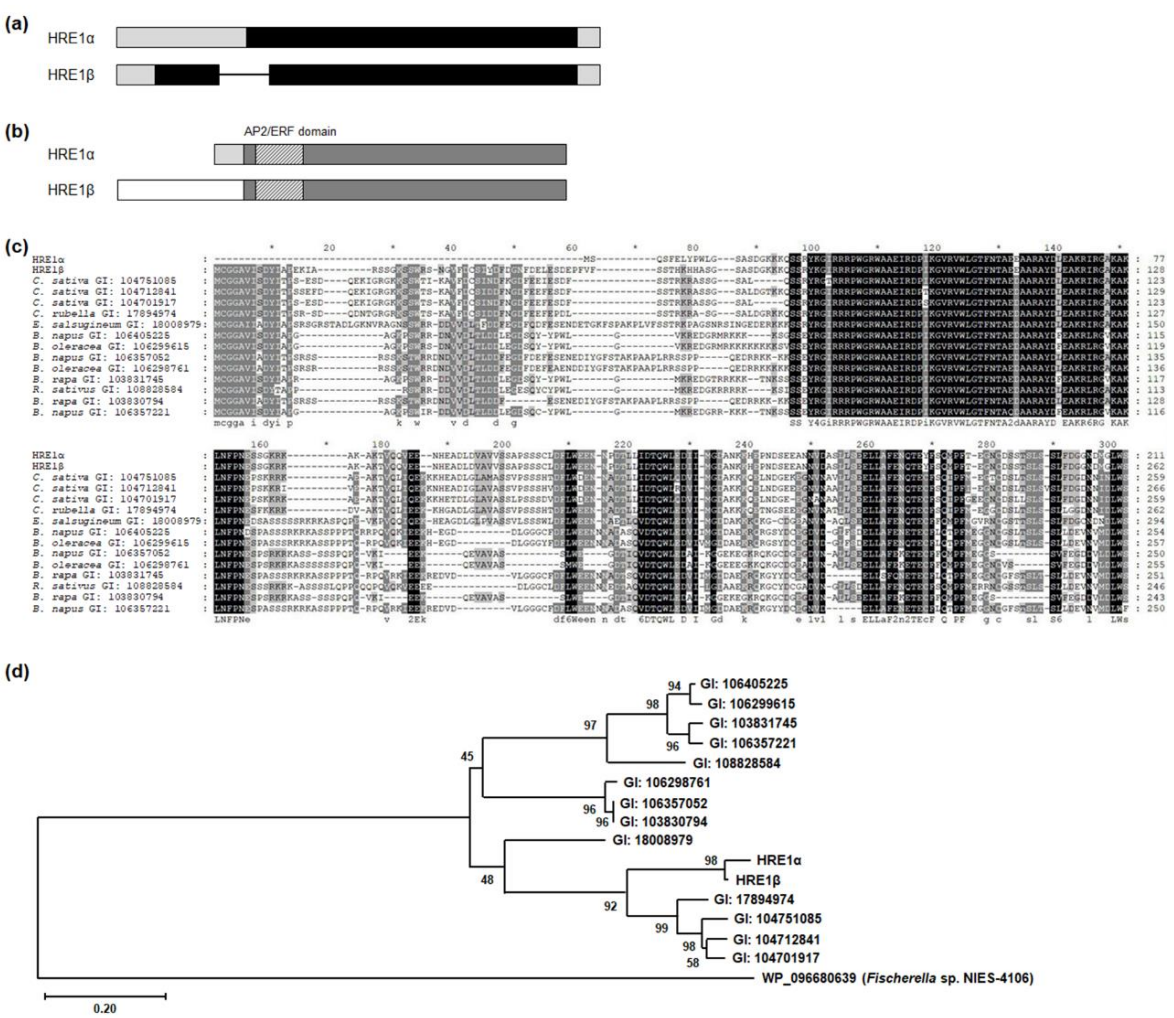
Splicing variant isoforms of HRE1. (**a**) The genomic structures of *HRE1α* and *HRE1β*. Black box, gray box, and line indicate coding region, untranslated region, and intron, respectively. (**b**) The protein domains of HRE1α and HRE1β. Dark gray box, light gray box, and white box indicate the common region of HRE1α and HRE1β, HRE1α-specific N-terminal region, and HRE1β-specific N-terminal region, respectively. Boxes with deviant crease line indicate the AP2/ERF domain. (**c**) Multiple alignment among HRE1α, HRE1β, and the orthologs of HRE1β in other plant species. Multiple alignment was performed with the amino acid sequences of the full-length ORFs of HRE1α, HRE1β, and the orthologs of HRE1β using Clustal Omega. (**d**) Phylogenetic tree of HRE1α, HRE1β, and the orthologs of HRE1β was generated with the full-length ORF. WP_096680639, an AP2 domain-containing protein from *Fischerella* sp. NIES-4109 was used as an outgroup.

Comparison of the genomic structures of *HRE1α* and *HRE1β* showed that *HRE1α* does not contain any intron, whereas *HRE1β* does have one (Figure 1a), and *HRE1α* and *HRE1β* encode proteins that potentially have 211 and 262 amino acids (aa), respectively (Figure 1b). HRE1α and HRE1β share 200 amino acids, including the AP2/ERF domain and C-terminal region, whereas the N-terminal region of HRE1β is longer than that of HRE1α because it harbors N-degron pathway-targeted sequences (Figure 1b) [35]. We performed BLASTP analysis to identify orthologs of both HRE1α and HRE1β in other plant species. Interestingly, no orthologs of HRE1α contained sequences similar to those in its N-terminal region. In contrast, all the orthologs corresponded to HRE1β, containing N-degron pathway-targeted sequences in their N-terminal regions (Figure 1c) [35]. In addition, HRE1α and HRE1β have no paralogs in *Arabidopsis*, whereas some of the other orthologs have paralogs in the same species (Figure 1c). We generated a phylogenetic tree to compare the phylogenetic relationship among HRE1α, HRE1β, and its orthologs. HRE1α and HRE1β were the two most closely related proteins and were close to the orthologs in *Camelina sativa* and *Capsella rubella* (Figure 1d).

### 2.2. Comparison of HRE1α and HRE1β Functions in the Hypoxia Response and Root Development

**Figure 2:**
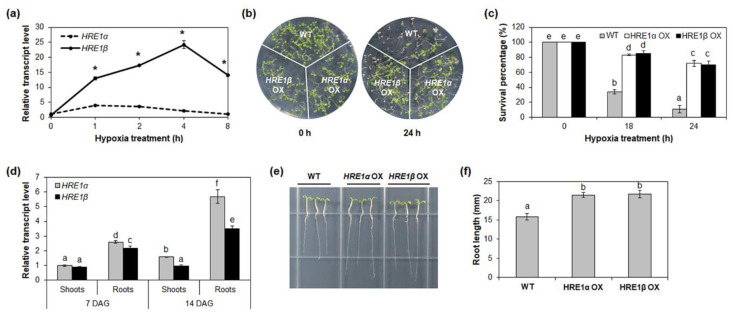
Hypoxia response and root development of *HRE1α* and *HRE1β*. (**a**) Quantitative RT-PCR analysis of *HRE1α* and *HRE1β* under hypoxia treatment for the indicated times. *GAPc* was used as an internal control. The transcript level of *HRE1α* at 0 h was set to 1. The reactions of each technical replicate were performed in triplicate. Two technical replicates were measured for each biological replicate. The data shown are the means ± S.D. (*n* = 6). Similar results were obtained from at least three biological replicates, with one shown here. * *t*-test *p* < 0.05. (**b**) Hypoxia response of *HRE1α* OXs and *HRE1β* OXs. Seven-day-old WT, *HRE1α* OXs, and *HRE1β* OXs were kept in the 99.99% nitrogen gas containing box under dark condition and then allowed to recover. (**c**) Survival percentages of WT, *HRE1α* OXs, and *HRE1β* OXs after hypoxia treatment and recovery. Thirty plants were used in each experiment. Three independent T_1_ lines of *HRE1α* OXs and *HRE1β* OXs showed very similar results, with one shown here. (**d**) Quantitative RT-PCR analysis of *HRE1α* and *HRE1β* in the shoots and roots of 7- and 14-day-old seedlings. *GAPc* was used as an internal control. The transcript level of *HRE1α* in the shoots of 7-day-old seedlings was set to 1. Reactions of each technical replicate were performed in triplicate. Two technical replicates were measured for each biological replicate. The data shown are the means ± S.D. (*n* = 6). Similar results were obtained from at least three biological replicates, with one shown here. (**e**) Primary roots of WT, *HRE1α* OXs, and *HRE1β* OXs. (**f**) The primary root length of 7-day-old WT, *HRE1α* OXs, and *HRE1β* OXs. Seven-day-old seedlings grown on MS plates under SD conditions were used. The data shown are the means ± S.D. (*n* = 15). Three independent T_1_ lines of *HRE1α* OXs and *HRE1β* OXs showed very similar results, with one shown here. In (**c**,**d**,**f**), the data were analyzed with one-way ANOVA using Tukey’s multiple comparison test. Different letters indicate significant differences (*p* < 0.05).

The expression of both *HRE1α* and *HRE1β* is upregulated by hypoxia [33,34]; to compare their expression patterns in response to such stress, the transcript levels of both splicing variants were analyzed under hypoxia conditions. Ten-day-old wild-type (WT) seedlings treated under low-oxygen conditions were analyzed by quantitative reverse-transcription PCR (RT-PCR) using *HRE1α* or *HRE1β*-specific primers (Table S1). As a result, the expression of both *HRE1α* and *HRE1β* was found to be higher after the hypoxia treatment, which was consistent with previous results (Figure 2a and Figure S1) [33,34]. The transcript levels of *HRE1β* were significantly higher than those of *HRE1α* under hypoxic conditions (Figure 2a).

Next, we examined the hypoxia responses of *HRE1α* OX and *HRE1β* OX seedlings. For this, *HRE1β* OXs were generated and selected using quantitative RT-PCR (Figure S2). The *HRE1α* OXs generated in previous study were used [33]. *HRE1α* OX, *HRE1β* OX, and WT seeds were sown onto the same plate and treated with hypoxia 7 days after germination (DAG). After 18 or 24 h of hypoxia treatment, both *HRE1α* OX and *HRE1β* OX seedlings were more tolerant than WT, with higher survival percentages (Figure 2b,c); we also found no significant phenotypic difference between the *HRE1α* OXs and *HRE1β* OXs (Figure 1b,c). Despite a previous report stating that *hre1* mutants do not show significant differences from the WT under hypoxia conditions [34], these results suggested that both *HRE1α* and *HRE1β* are involved in the hypoxia response.

In a previous study, we showed that *HRE1α* is involved in primary root elongation via regulation of root meristem cell division [33]. In addition, the expression level of *HRE1β* has been shown to be higher in the roots than in the shoots [34]. To compare the functions of *HRE1α* and *HRE1β* in root development, we analyzed their expression in the shoots and roots using 7- and 14-day-old WT seedlings. The expression levels were similar in the shoots of both 7- and 14-day-old seedlings (Figure 2d). However, the expression level of *HRE1α* was significantly higher than that of *HRE1β* in the roots (Figure 2d).

We next compared the root lengths of the *HRE1α* and *HRE1β* OXs. For this, the *HRE1α* and *HRE1β* OX seeds were sown onto the same Murashige and Skoog (MS) plates as the WT seeds and the primary root lengths of the seedlings were measured at 7 DAG. Both of the OXs had longer primary roots than the WT plants (Figure 2e,f), and the primary root length did not significantly differ between the *HRE1α* OXs and *HRE1β* OXs (Figure 2e,f). These results suggest that both splicing variants function in primary root development. However, *hre1* mutants did not show significant difference in root elongation compared to WT (data not shown).

### 2.3. N-Terminal and/or C-Terminal Regions of HRE1α and HRE1β Are Responsible for Transactivation Activity

**Figure 3:**
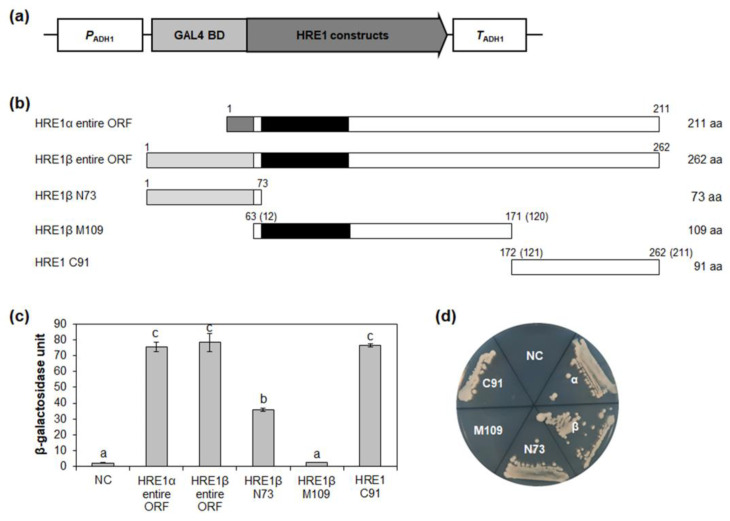
Analysis of transactivation activities of HRE1α and HRE1β. (**a**) The schematic map of the vector for analysis of transactivation activities of full-length ORFs of HRE1α and HRE1β, and truncated fragments of HRE1β in yeast. (**b**) The schematic maps of full-length ORFs of HRE1α and HRE1β, and truncated fragments of HRE1β for the analysis of transactivation activity in yeast. The numbers in parentheses represent the position in HRE1α. (**c**) Quantitative β-galactosidase ONPG assay. The transactivation activities were quantified by measuring the β-galactosidase activity in yeast extract. The experiments of each technical replicate were performed in triplicate. The data shown are the means ± S.D. (*n* = 3). Similar results were obtained from at least three biological replicates, with one shown here. The data were analyzed with one-way ANOVA using Tukey’s multiple comparison test. Different letters indicate significant differences (*p* < 0.05). (**d**) Yeast growth assay. Yeast transformants were grown on SM-Trp/-Ura. In (**c**,**d**), pBD-GAL4 vector itself was used as a negative control.

Previously, we reported that HRE1α has transactivation activity and that its C-terminal region is responsible for this activity [33]. However, the same was not previously investigated for HRE1β and was thus investigated in this study using the yeast system. As a result, we found that the full-length HRE1β showed transactivation activity in yeast, which was very similar to that of the full-length HRE1α (Figure 3c,d and Figure S3a). These results indicated that both HRE1β and HRE1α function as transcriptional activators.

To identify the domain responsible for its transactivation activity, HRE1β was divided into three regions: the N-terminal 1–73-aa region (N73), middle 63–171-aa region (M109), and C-terminal 172–262-aa region (C91) (Figure 3b). In the quantitative β-galactosidase 2-nitrophenyl-β-D-galactopyranoside (ONPG) assay and yeast growth assay, C91 showed the highest activity among the three regions, almost the same as that of the full-length HRE1β (Figure 3c,d). N73 also showed transactivation activity, although it was weaker than that of C91 (Figure 3c,d). However, M109, containing the AP2/ERF DNA-binding domain, did not show transactivation activity (Figure 3c,d). These results indicated that both the N-terminal and C-terminal regions of HRE1β contain domains with transactivation activity, unlike HRE1α, in which only the C-terminal region shows activity.

### 2.4. N-Terminal Region of HRE1β Has One Motif Responsible for Transactivation Activity

**Figure 4:**
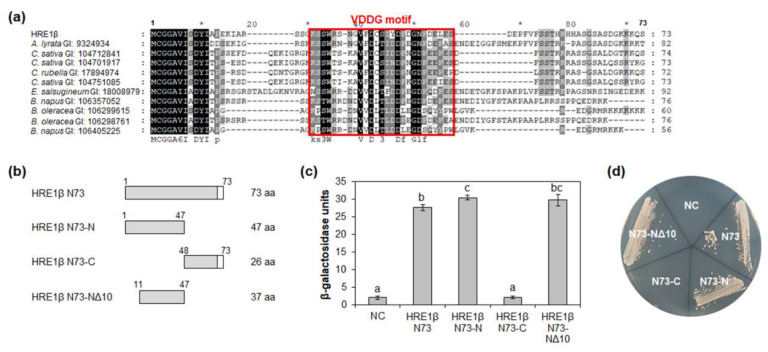
Analysis of transactivation activity of N-terminal region of HRE1β. (**a**) Multiple sequence alignment was carried out with the amino acid sequences of HRE1β N73 and corresponding regions of its orthologs using Clustal Omega. The GI number of each protein sequence is as follows: *Arabidopsis lyrata*: 9324934; *Camelina sativa*: 104712841, 104701917, 104751085; *Capsella rubella*: 17894974; *Eutrema salsugineum*: 18008979; *Brassica napus*: 106357052, 106405225; *Brassica oleracea*: 106299615, 106298761. The VDDG motif is represented as a box. (**b**) The schematic maps of HRE1β N73 and its partial fragments for the analysis of transactivation activity in yeast. (**c**) Quantitative β-galactosidase ONPG assay. The transactivation activities were quantified by measuring the β-galactosidase activity in yeast extract. The experiments of each technical replicate were performed in triplicate. The data shown are the means ± S.D. (*n* = 3). Similar results were obtained from at least three biological replicates, with one shown here. The data were analyzed with one-way ANOVA using Tukey’s multiple comparison test. Different letters indicate significant differences (*p* < 0.05). (**d**) Yeast growth assay. Yeast transformants were grown on SM-Trp/-Ura. In (**c**,**d**), pBD-GAL4 vector itself was used as a negative control.

We identified the orthologs of HRE1β N73 in other plant species, including *Arabidopsis lyrata*, *Camelina sativa*, *Capsella rubella*, *Eutrema salsugineum*, *Brassica napus*, and *Brassica oleracea*, using the BLASTP tool. Multiple alignment among HRE1β N73 and its orthologs was performed using the Clustal Omega tool. Forty-seven amino acids in HRE1β N73, corresponding to the 1–47-aa region in HRE1β, were highly conserved among HRE1β and its orthologs (Figure 4a). The first 10 amino acids in this sequence, MCGGAVISDY, are known to be related to the N-degron pathway, which is involved in the protein degradation process [35].

To identify the motif(s) responsible for transactivation activity in HRE1β N73, partial fragments of HRE1β N73, namely, N73-N (1–47 aa), N73-C (48–73 aa), and N73-NΔ10 (11–47 aa; N73-N without 1–10 aa), were fused to GAL4 DNA-binding domain (BD) (Figure 4b). In the quantitative β-galactosidase ONPG assay and yeast growth assay, both N73-N and N73-NΔ10 showed transactivation activity, while N73-C did not (Figure 4c,d). In addition, there was no significant difference in the activity between N73-N and N73-NΔ10 (Figure 4c,d), indicating that the 37-aa sequence of N73-NΔ10 is important for transactivation activity. We named the highly conserved 26-aa sequence in the N73-NΔ10 (22–47 aa) as the VDDG motif (Figure 4a).

### 2.5. C-Terminal Region of HRE1β Has Two Motifs Responsible for Transactivation Activity

**Figure 5:**
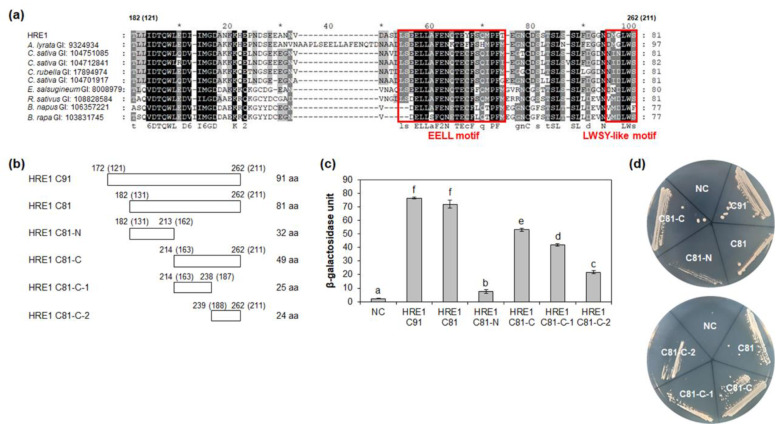
Analysis of transactivation activity of C-terminal region of HRE1β. (**a**) Multiple sequence alignment was carried out with the amino acid sequences of HRE1β C81 and corresponding regions of its orthologs using Clustal Omega. The GI number of each protein sequence is as follows: *Arabidopsis lyrata*: 9324934; *Camelina sativa*: 104751085, 104712841, 104701917; *Capsella rubella*: 17894974; *Eutrema salsugineum*: 18008979; *Raphanus sativus*: 108828584; *Brassica napus*: 106357221; *Brassica rapa*: 103831745. The EELL motif and LWSY-like motif are represented as boxes. (**b**) The schematic maps of HRE1β C91 and C81, and partial fragments of C81 for the analysis of transactivation activity in yeast. The numbers in parentheses represent the position in HRE1α. (**c**) Quantitative β-galactosidase ONPG assay. The transactivation activities were quantified by measuring the β-galactosidase activity in yeast extract. The experiments of each technical replicate were performed in triplicate. The data shown are the means ± S.D. (*n* = 3). Similar results were obtained from at least three biological replicates, with one shown here. The data were analyzed with one-way ANOVA using Tukey’s multiple comparison test. Different letters indicate significant differences (*p* < 0.05). (**d**) Yeast growth assay. Yeast transformants were grown on SM-Trp/-Ura. In (**c**,**d**), pBD-GAL4 vector itself was used as a negative control.

We next analyzed the motif(s) in the C-terminal region of HRE1α/HRE1β responsible for transactivation activity. First, we compared the activity of HRE1β C91 (172–262 aa) with that of HRE1β C81 (182–262 aa) because the 121–130-aa region of HRE1α corresponding to the 172–181-aa region of HRE1β has been shown to be unimportant for the activity [33]. Quantitative β-galactosidase ONPG and yeast growth assays revealed that both C91 and C81 show transactivation activity, with no significant difference between the activities of these two regions (Figure 5c,d).

We further analyzed the motif(s) responsible for transactivation activity in HRE1β C81. BLASTP analysis using HRE1β C81 identified orthologs in other plant species, such as *Arabidopsis lyrata*, *Camelina sativa*, *Capsella rubella*, *Eutrema salsugineum*, *Raphanus sativus*, *Brassica napus*, and *Brassica rapa*, and multiple alignment between them was performed (Figure 5a). Based on the conservation of amino acid sequences, HRE1β C81 was divided into two subregions: HRE1β C81-N (182–213 aa) and HRE1β C81-C (214–262 aa) (Figure 5b). The results of quantitative β-galactosidase ONPG and yeast growth assays showed that both C81-N and C81-C exhibited transactivation activity, and the activity of C81-N was lower than that of C81-C (Figure 5c,d).

To narrow the possible motif(s) responsible for transactivation activity, HRE1β C81-C was divided into two subregions: C81-C-1 (214–238 aa) and C81-C-2 (239–262 aa) (Figure 5b). The results of quantitative β-galactosidase ONPG assay and yeast growth assay showed that both C81-C-1 and C81-C-2 exhibited transactivation activity, with that of C81-C-1 being higher than that of C81-C-2 (Figure 5c,d). These results suggested that both the C81-C-1 and C81-C-2 contain motif(s) are responsible for transactivation activity.

### 2.6. Four Glutamate Residues and LWS Residues Contribute to Transactivation Activity

**Figure 6:**
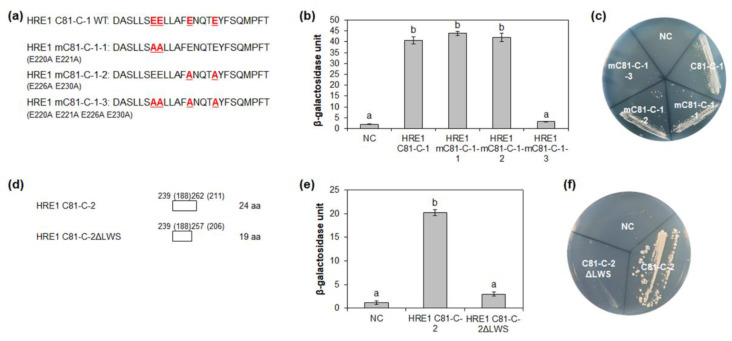
Identification of the amino acid residues responsible for transactivation activity. (**a**) The amino acid sequences of HRE1β C81-C-1 and its site-directed mutated sequences for the analysis of transactivation activity in yeast. (**b**,**e**) Quantitative β-galactosidase ONPG assay. The transactivation activities were quantified by measuring the β-galactosidase activity in yeast extract. The experiments of each technical replicate were performed in triplicate. The data shown are the means ± S.D. (*n* = 3). Similar results were obtained from at least three biological replicates, with one shown here. The data were analyzed with one-way ANOVA using Tukey’s multiple comparison test. Different letters indicate significant differences (*p* < 0.05). (**c**,**f**) Yeast growth assay. Yeast transformants were grown on SM-Trp/-Ura. (**d**) The schematic maps of HRE1β C81-C-2 and its partial fragment for the analysis of transactivation activity in yeast. The numbers in parentheses represent the position in HRE1α. In (**b**,**c**,**e**,**f**), pBD-GAL4 vector itself was used as a negative control.

It is well-known that specific acidic aa residues, such as D and E, are important for transactivation activity [12]. Four E residues (E220, E221, E226, and E230) in HRE1β C81-C-1 were highly conserved among HRE1β and its orthologs (Figure 5a). To assess their importance for the transactivation activity of C81-C-1, each E residue was replaced with an alanine (A) residue by point mutation, resulting in mC81-C-1-1 (E220A E221A), mC81-C-1-2 (E226A E230A), and mC81-C-1-3 (E220A E221A E226A E230A) (Figure 6a). Quantitative β-galactosidase ONPG and yeast growth assays showed that mC81-C-1-1 and mC81-C-1-2 showed similar activity to the nonmutated C81-C-1, whereas mC81-C-1-3 showed almost no activity (Figure 6b,c). These results indicated that all four E residues (E220, E221, E226, and E230) in the C-terminal region of HRE1β are necessary for its transactivation activity.

The LWSY motif of RAP2.12 and RAP2.2 in *Arabidopsis*, which belong to group VII of the ERF subfamily, is important for the transactivation activity of these transcription factors [8]. HRE1β C81-C-2 also has amino acid sequences similar to the LWSY motif at its C-terminal end; the sequences were found to be highly conserved among HRE1β and its orthologs (Figure 5a). To analyze whether LWSY-like sequences are important for transactivation activity, C81-C-2 without LWSY-like sequences (C81-C-2ΔLWS) was fused to GAL4 BD (Figure 6d). The results of quantitative β-galactosidase ONPG and yeast growth assays showed that C81-C-2ΔLWS had significantly lower activity than C81-C-2 (Figure 6e,f), suggesting that LWSY-like sequences consisting of five amino acids in HRE1β C81-C-2 contribute to the transactivation activity. We named the 21-aa sequence of C81-C-1 (218–238 aa) as the EELL motif and the five amino acids of C81-C-2 (MGLWS) as the LWSY-like motif (Figure 5a).

### 2.7. HRE1α and HRE1β Show Transactivation Activity In Vivo

**Figure 7:**
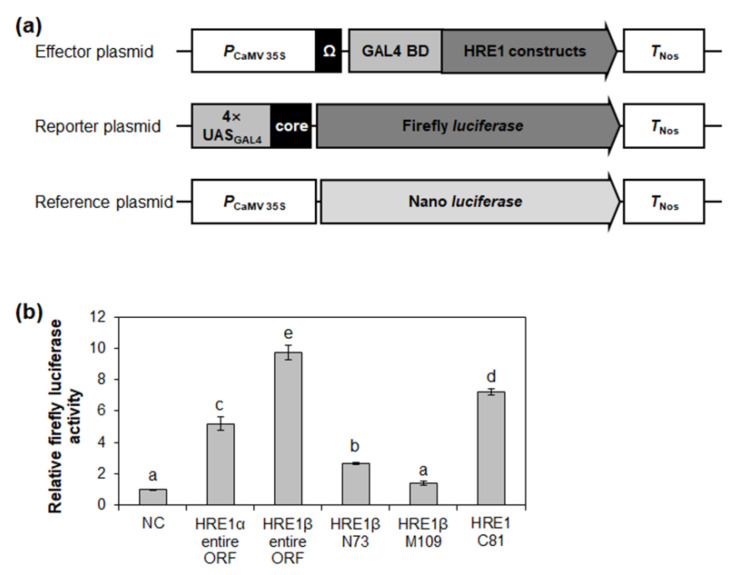
Analysis of transactivation activities of HRE1α and HRE1β. (**a**) The schematic maps of the effector plasmid, reporter plasmid, and reference plasmid for the analysis of transactivation activities of full-length ORFs of HRE1α and HRE1β, and truncated fragments of HRE1β. (**b**) The relative firefly luciferase activity in *Arabidopsis* protoplasts. The transformation efficiency was normalized with Nano luciferase activity of the reference plasmid. The normalized firefly luciferase activity of negative control was set as 1. The empty effector plasmid was used as a negative control. The data shown are the means ± S.D. (*n* = 3). The data were analyzed with one-way ANOVA using Tukey’s multiple comparison test. Different letters indicate significant differences (*p* < 0.05).

Transactivation assay systems including effector, reporter, and reference plasmids, have been widely used to compare the transactivation activities of proteins and/or their domains in plant protoplasts [36,37,38]. To confirm the transactivation activity of HRE1α and HRE1β in *Arabidopsis*, effector plasmids, in which each full-length open reading frame (ORF) of HRE1α and HRE1β was fused to GAL4 BD, were generated and transformed into *Arabidopsis* protoplasts (Figure 7a). Transient expression of the effector plasmid of HRE1α or HRE1β with the reporter plasmid, harboring the firefly luciferase gene under the control of 4× GAL4 upstream activating sequence, showed that the full-length ORFs of both HRE1α and HRE1β exhibit transactivation activity in *Arabidopsis* protoplasts (Figure 7b). The activity of HRE1β was found to be higher than that of HRE1α in *Arabidopsis* protoplasts (Figure 7b).

The transactivation activity of HRE1β N73, HRE1β M109, and HRE1β C81 was also assessed in *Arabidopsis* protoplasts. HRE1β C81 showed strong activity in *Arabidopsis* protoplasts, while HRE1β N73 showed weak activity, and HRE1β M109 did not (Figure 7b). These results were similar to those found in yeast (Figure 3).

### 2.8. Both HRE1α and HRE1β Transactivate Downstream Genes via the GCC Box

**Figure 8:**
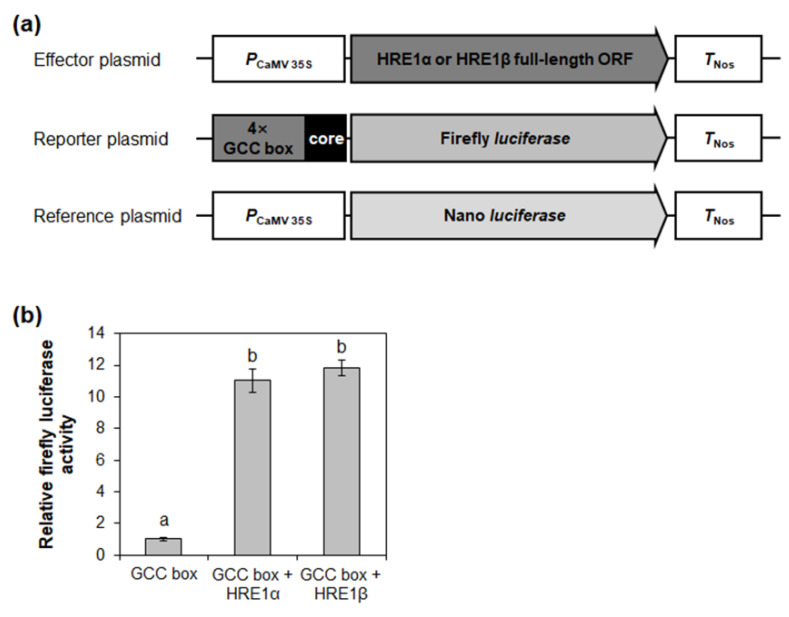
Transactivation assay of HRE1α and HRE1β via GCC box. (**a**) The schematic maps of the effector plasmid, reporter plasmid, and reference plasmid for the transactivation assay of HRE1α and HRE1β via GCC box. (**b**) The relative firefly luciferase activity in *Arabidopsis* protoplasts. The transformation efficiency was normalized with Nano luciferase activity of the reference plasmid. The normalized firefly luciferase activity of negative control was set as 1. The empty effector plasmid was used as a negative control. The data shown are means ± S.D. (*n* = 3). The data were analyzed with one-way ANOVA using Tukey’s multiple comparison test. Different letters indicate significant differences (*p* < 0.05).

It was previously shown that HRE1α transactivates downstream genes via the GCC box [33]. To investigate whether HRE1β also regulates downstream genes via the GCC box, effector plasmids for the expression of HRE1α and HRE1β were transformed into *Arabidopsis* protoplasts with reporter plasmids containing the firefly luciferase gene under the control of 4× GCC box core sequences (Figure 8a). Transactivation assay results showed a great relative increase of reporter activity by both HRE1α and HRE1β (Figure 8b), suggesting that both transcription factors transactivate downstream genes via the GCC box. Recently, it was reported that hypoxia-responsive promoter element (HRPE) is a *cis*-acting element regulated by members from group VII of the ERF subfamily, including *HRE1*, *HRE2*, *RAP2.12*, *RAP2.2*, and *RAP2.3* [39]. However, we did not ascertain whether HRE1α and HRE1β transactivate downstream genes via HRPE because HRE1 (HRE1β) does not bind to HRPE [39].

### 2.9. Analysis of Downstream Genes Regulated by HRE1α and HRE1β Using RNA-Seq

To identify downstream genes regulated by HRE1α and HRE1β, RNA-Seq analysis was performed using *HRE1α* OXs, *HRE1β* OXs, and WT transcripts. The mapping of RNA-Seq reads to the Columbia genome was successful, with mapping rates of 96.3–98.0% (Table S2). The number of mapped reads ranged from 26.7 to 31.2 million (Table S2). The less abundant genes were removed, and finally, a total of 22,884 genes were included in the analysis.

**Figure 9:**
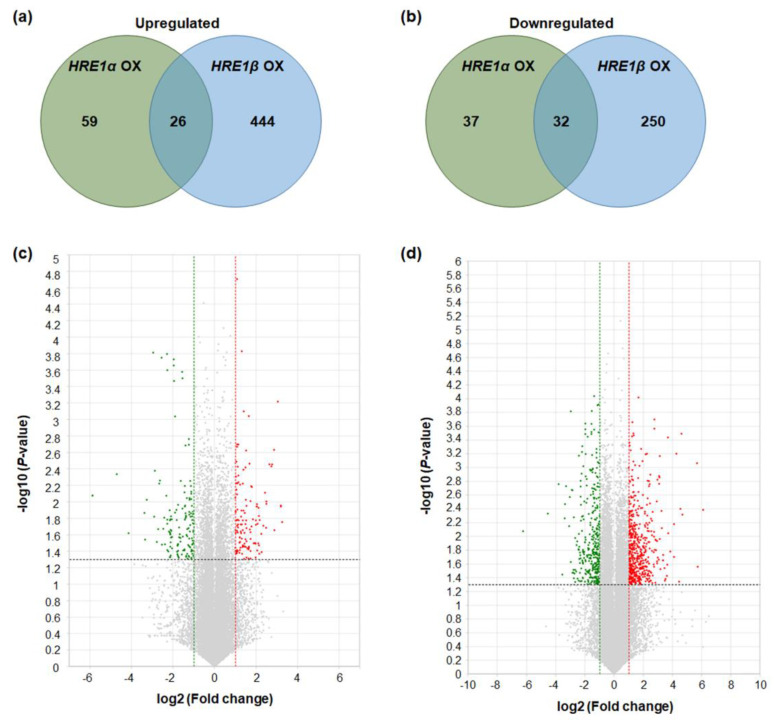
Differentially expressed genes in *HRE1α* OXs and/or *HRE1β* OXs. (**a**) The numbers of genes that were upregulated in *HRE1α* OXs and/or *HRE1β* OXs relative to those in WT plants with >2-fold differences in expression and a *p* < 0.05. (**b**) The numbers of genes that were downregulated in *HRE1α* OXs and/or *HRE1β* OXs relative to those in WT plants with >2-fold differences in expression and a *p* < 0.05. (**c**) Volcano plot of DEGs identified between WT and *HRE1α* OXs. (**d**) Volcano plot of DEGs identified between WT and *HRE1β* OXs. In (**c**) and (**d**), DEGs were selected by *p* < 0.05 and log2 ratio ≥ 1 conditions.

Genes with >2-fold differences in expression, with *p* < 0.05, were considered to be up- or downregulated in the *HRE1α* OXs and/or *HRE1β* OXs. The results showed that 85 and 470 genes were upregulated in *HRE1α* OXs and *HRE1β* OXs, respectively (Figure 9a). Among these, 26 genes were upregulated in both the *HRE1α* OXs and *HRE1β* OXs (Figure 9a). In contrast, 69 and 282 genes were downregulated in *HRE1α* OXs and *HRE1β* OXs, respectively (Figure 9b). Among these, 32 genes were downregulated in both the *HRE1α* OXs and *HRE1β* OXs (Figure 9b). The distribution of these differentially regulated genes is shown using volcano plots (Figure 9c,d). These results indicated that HRE1α and HRE1β could differentially transactivate downstream genes, and HRE1β could transactivate more genes than HRE1α.

**Figure 10:**
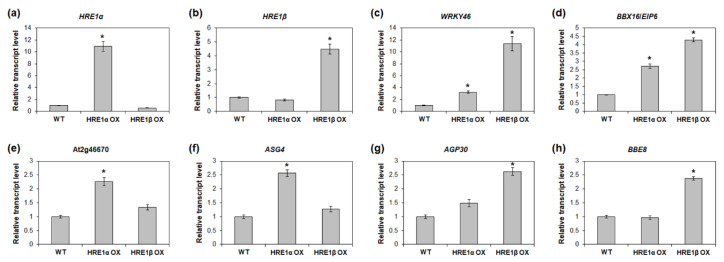
Expression analysis of downstream genes of *HRE1α* and/or *HRE1β* in *HRE1α* OXs and *HRE1β* OXs. Quantitative RT-PCR analysis of *HRE1α* (**a**), *HRE1β* (**b**), *WRKY46* (**c**), *BBX16*/*EIP6* (**d**), At2g46670 (**e**), *ASG4* (**f**), *AGP30* (**g**), and *BBE8* (**h**) in WT, *HRE1α* OX, and *HRE1β* OX seedlings. *GAPc* was used as an internal control. The transcript levels in WT were set as 1. Three independent reactions were performed for each technical replicate. Two technical replicates were performed for each biological replicate. At least two biological replicates showed similar results, with one shown here. The data shown are the means ± S.D. (*n* = 6) and * indicates *t*-test *p* < 0.05.

To validate the RNA-Seq results, we analyzed the expression of the genes upregulated in *HRE1α* OXs and/or *HRE1β* OXs. First, we confirmed that the expression of *HRE1α* and *HRE1β* was upregulated in the *HRE1α* OXs and *HRE1β* OXs, respectively (Figure 10a,b). We selected the upregulated genes, namely, *WRKY46*, *BBX16*/*EIP6*, At2g46670, *ASG4*, *AGP30*, and *BBE8*, in *HRE1α* OXs and/or *HRE1β* OXs from the RNA-Seq analysis results (Tables S3 and S4) for use in expression analysis. The results showed that *WRKY46* and *BBX16*/*EIP6*, which were upregulated in both the *HRE1α* OXs and *HRE1β* OXs, had higher expression levels in both the *HRE1α* OXs and *HRE1β* OXs than in the WT plants (Figure 10c,d). Expression levels of At2g46670 and *ASG4*, which were upregulated only in *HRE1α* OXs, were confirmed to be higher in *HRE1α* OXs than in WT plants; no significant difference in their expression was detected between *HRE1β* OXs and WT plants (Figure 10e,f). The expression levels of *AGP30* and *BBE8*, which were upregulated in *HRE1β* OXs, were higher in *HRE1β* OXs than in WT plants; no significant difference in the expression levels of these genes was detected between *HRE1α* OXs and WT plants (Figure 10g,h). These results were consistent with the RNA-Seq results.

Gene ontology (GO) analysis is useful to predict the biological and molecular processes altered by changes in gene expression patterns. We further analyzed the biological process GO annotation categories of the differentially expressed genes (DEGs) in *HRE1α* OXs and *HRE1β* OXs to reveal their biological functions. The genes upregulated in *HRE1α* OXs, but not in *HRE1β* OXs, were enriched in the regulation of biological processes such as flower development, shoot system development, circadian rhythm, gene expression regulation, response to gibberellin, nitrogen compound metabolic process, and aromatic compound biosynthetic process (Table 1). However, the genes upregulated in *HRE1β* OXs, but not in *HRE1α* OXs, were enriched in the regulation of the following processes: response to toxic substance, secondary metabolic process, response to biotic stimulus, glutathione metabolic process, response to reactive oxygen species, cell wall organization, DNA replication, response to salicylic acid, lipid localization, response to oxidative stress, carbohydrate metabolic process, photosynthesis, mitotic cell cycle process, response to osmotic stress, and response to abscisic acid (Table 2). These results suggested that HRE1α and HRE1β might transactivate genes in different downstream pathways. The genes upregulated in both *HRE1α* OXs and *HRE1β* OXs were enriched in biological processes involved in response to abiotic stimulus, response to light stimulus, response to endogenous stimulus, response to oxygen-containing compound, and response to lipid (Table 1 and Table 2), indicating that both *HRE1α* and *HRE1β* might be involved in abiotic stress responses.

We further analyzed the downregulated genes in the *HRE1α* OXs and *HRE1β* OXs. Genes downregulated in *HRE1α* OXs, but not in *HRE1β* OXs, were enriched in the regulation of biological processes, such as response to salicylic acid, response to jasmonic acid, cellular response to acid chemical, proteasomal protein catabolic process, response to lipid, response to nitrogen compound, and regulation of macromolecule biosynthetic process (Table 3). However, genes downregulated in *HRE1β* OXs, but not in *HRE1α* OXs, were enriched in the regulation of biological processes such as response to abiotic stimulus, plant-type cell wall organization, phosphorelay signal transduction system, response to radiation, response to brassinosteroid, hydrogen peroxide catabolic process, and cellular response to ethylene stimulus (Table 4). The genes downregulated in both OXs were enriched in biological processes involved in response to endogenous stimulus, response to organic substance, response to hormone, response to oxygen-containing compound, and regulation of transcription (Table 3 and Table 4). These results indicated that HRE1α and HRE1β might be involved in both similar and different signal transduction pathways.

### 2.10. Analysis of Genes Downstream of HRE1α and HRE1β in the Hypoxia Response

To characterize the regulation of responses to hypoxia by *HRE1α* and *HRE1β*, the expression of the 85 upregulated genes in *HRE1α* OXs and the 470 upregulated genes in *HRE1β* OXs under hypoxic conditions was analyzed using Genevestigator. Two anoxia experiments and eight hypoxia experiments were used for this analysis. Among the 85 genes upregulated in *HRE1α* OXs, the expression of four genes, namely, *APRR9*, *WRKY46*, *CNGC12*, and *RAB18*, increased under conditions of anoxia and hypoxia (Figure S4). Among the 470 upregulated genes in *HRE1β* OXs, the expression of 65 genes, including *HSP17.6*, *CYP81D11*, *UGT73B*, *SAP12*, *ZAT12*, *GSTU1*, and *ANAC032*, increased under conditions of anoxia and hypoxia (Figure S5). The four genes in *HRE1α* OXs and 65 genes in *HRE1β* OXs corresponded to 66 individual genes, which included seven glutathione S-transferase (GST) genes, four UDP-glucosyltransferase (UGT) genes, four cytochrome P450 (CYP) genes, and four WRKY genes (Figures S4 and S5). These results suggested that these genes could be involved in responses to hypoxia mediated by *HRE1α* and/or *HRE1β*, and that HRE1β could transactivate more hypoxia-responsive genes than HRE1α.

## 3. Discussion

Alternative splicing of pre-mRNAs allows organisms to increase their coding potential and is one of various stress response mechanisms in plants [18]. HRE1 is an *Arabidopsis* AP2/ERF transcription factor, and its gene has two alternative splicing variants, *HRE1α* and *HRE1β* [33]; both are involved in the response to hypoxia in *Arabidopsis* [33,34]. However, their functions have not yet been compared and studied. In this study, we describe the differences between the functions of *HRE1α* and *HRE1β* in the hypoxia response and root development of *Arabidopsis*.

Our results showed that both *HRE1α* and *HRE1β* are involved in hypoxia response and root development. RT-PCR analysis results showed that the expression of both *HRE1α* and *HRE1β* increased under hypoxia conditions (Figure 2a and Figure S1). *HRE1β* was expressed at significantly higher levels than *HRE1α* under hypoxia (Figure 2a and Figure S1), indicating that *HRE1β* might have a more important role than *HRE1α* in the hypoxia response. In addition, both *HRE1α* and *HRE1β* showed higher expression levels in the roots than in the shoots (Figure 2d), and the primary root length in both OXs was greater than that of the WT seedlings (Figure 2e,f). *HRE1α* showed higher expression levels in the roots than *HRE1β* (Figure 2d), implying that *HRE1α* plays more important roles than *HRE1β* in root development although both *HRE1α* and *HRE1β* are involved in root development.

BLASTP and multiple alignment analyses of HRE1 orthologs showed highly conserved sequences in the N-terminal region of HRE1β, but not of HRE1α (Figure 1c), demonstrating that *HRE1α* might have evolved after *Arabidopsis* diverged from related plant species. In addition, comparison of protein sequences showed that HRE1α and HRE1β share the same amino acid sequences in their AP2/ERF domain and C-terminal regions, whereas they have distinct N-terminal sequences (Figure 1). While HRE1β has N-degron pathway-targeted sequences, HRE1α does not (Figure 1) [35]. This result suggests that HRE1α might not be a target of the N-degron pathway, therefore, HRE1α might be more stable than HRE1β as it is protected from N-degron targeted protein degradation under normal conditions.

The well conserved transactivation motifs have been identified in transcription factors in plants. AHA motif present in HD-Zip I family proteins, such as AtHB1, AtHB7, AtBH12, and AtHB13, has transactivation activity [40]. EELR motif functions as a transactivation motif in a CCCH zinc finger protein, OsLIC [41]. In AP2/ERF transcription factor family proteins, transactivation motifs are not well identified, yet, except LWSY motif and EDLL motif [8,42]. In this study, we identified three novel transactivation motifs in AP2/ERF transcription factors, HRE1α and HRE1β. HRE1α was previously identified to be a transcriptional activator, and its C-terminal region plays an important role in its transactivation activity [33]; however, the motif in the C-terminal region responsible for this was not previously identified. In this study, we confirmed that both HRE1β and HRE1α show transactivation activity (Figure 3 and Figure 7 and Figure S3a), having the same amino acid sequences in their AP2/ERF domain and C-terminal region (Figure 1b,c). Further analysis of the C-terminal region showed that two motifs, the EELL and LWSY-like motifs, are responsible for the transactivation activity of HRE1α/HRE1β (Figure 5 and Figure 6 and Figure S3c,d). Specifically, all four E residues in the EELL motif play major roles (Figure 6b,c and Figure S3d). The LWSY-like motif consists of MGLWS amino acids, similar to the LWSY motif (Figure 5a). The LWSY motif has been identified in several ERF and DREB/CBF proteins, such as RAP2.12 and RAP2.2, which are members of group VII of the ERF subfamily. Both RAP2.12 and RAP2.2 contain an LWSY motif at the end of their C-terminal region, and the LWSY motif in RAP2.12 and RAP2.2 plays an important role in their transactivation activity [8]. The N-terminal region of HRE1β showed transactivation activity due to its VDDG motif, whereas that of HRE1α did not (Figure 3, Figure 4, and Figure 7 and Figure S3b). As HRE1β has one more motif involved in transactivation activity than HRE1α, we expected that HRE1β might show stronger transactivation activity than HRE1α. Indeed, the transactivation activity of HRE1β was stronger than that of HRE1α in *Arabidopsis* protoplasts (Figure 7), indicating that HRE1β might be more strongly involved in transcriptional regulation than HRE1α. The VDDG, EELL, and LWSY-like motifs are highly conserved among HRE1β and its orthologs (Figure 4a and Figure 5a). To our knowledge, this study is the first to propose that the VDDG, EELL, and LWSY-like motifs represent transactivation motifs.

RNA-Seq analysis using *HRE1α* OXs and *HRE1β* OXs revealed that HRE1β transactivates more downstream genes than HRE1α (Figure 9). We speculate that this differential transactivation could be caused by the stronger transactivation activity of HRE1β and the difference in overexpression levels of *HRE1α* and *HRE1β* in their OXs (Figure 7 and Figure 10). The results of this analysis showed that both splicing variants are involved in the regulation of abiotic stress responses (Table 1 and Table 2). In addition, we found that *HRE1α* regulates developmental processes and metabolism of nitrogenous and aromatic compounds, whereas *HRE1β* is involved in biotic stress responses, cell cycle regulation, carbohydrate metabolism, and photosynthesis (Table 1 and Table 2). These results suggested that both *HRE1α* and *HRE1β* might function in abiotic stress responses, while they also transactivate different downstream pathways. Although they both have the same AP2/ERF DNA-binding domain and bind to the same *cis*-acting element, the GCC box (Figure 8), we could not confirm whether the direct or the downstream target genes of HRE1α and HRE1β are the same. Recently, it was reported that RAP2.12 and RAP2.2, members of group VII of the ERF subfamily, bind to HRPE and directly transactivate *LBD41* and *PCO1* containing HRPE in their promoters whereas HRE1 (HRE1β) does not bind to HRPE [39], suggesting that downstream pathway of HRE1 would be different to that of RAP2.12 and RAP2.2.

Genevestigator analysis using genes upregulated in *HRE1α* OXs and *HRE1β* OXs showed that four genes in *HRE1α* OXs and 65 genes in *HRE1β* OXs, corresponding to 66 individual genes, were upregulated under hypoxia conditions (Figures S4 and S5). This suggested that these 66 genes might be involved in the hypoxia response of HRE1α and/or HRE1β, and also that HRE1β transactivates more hypoxia-responsive genes than HRE1α. This result, together with the higher expression of *HRE1β* under hypoxic stress, suggests that *HRE1β* plays a more important role than *HRE1α* in the hypoxia response of *Arabidopsis*.

Taken together, our study revealed that HRE1α and HRE1β, the two alternative splicing variants of HRE1, differentially transactivate downstream genes in the hypoxia response and root development of *Arabidopsis*.

## 4. Materials and Methods

### 4.1. Plant Materials and Growth Conditions

All *Arabidopsis thaliana* plants used in this study were of the Columbia ecotype. For surface-sterilization, seeds were dipped for 1 min in 70% ethanol, followed by dipping in 10 min in 1/10-diluted commercial bleach (0.4% NaOCl); then, they were washed with sterile distilled water four times. The seeds were placed in the dark for 2 days at 4 °C, and the seedlings were grown on agar plates containing salts and vitamins in half-strength MS medium [43], 2.0% sucrose, and 0.7% agar under short-day conditions (8/16 h light/dark cycles) at 22 °C. Ten-day-old seedlings were transferred to soil and grown under long-day conditions (16/8 h light/dark cycles) at 22 °C.

### 4.2. Plasmid Construction

To generate an *HRE1β*-overexpressing construct, the full-length ORF of *HRE1β* was amplified by PCR and then cloned into the *Bam*HI-*Xba*I sites in a binary vector, pFGL571 [44]. The *HRE1α* OXs used in this study are from the line employed in a previous study [33].

To generate constructs for the analysis of transactivation activity in yeast, full-length ORFs and partial fragments of *HRE1α* and *HRE1β* were amplified by PCR and cloned into the *Eco*RI-*Sal*I sites in pBD-GAL4 in frame with GAL4 BD. To generate constructs for the analysis of the transactivation activity in *Arabidopsis* protoplasts, full-length ORFs and partial fragments of *HRE1α* and *HRE1β* were fused with GAL4 BD under the control of cauliflower mosaic virus (CaMV) 35S promoter.

The primers used for cloning are listed in Table S5.

### 4.3. Plant Transformation and Selection of Transgenic Plants

The binary vectors were introduced into *Agrobacterium tumefaciens* strain GV3101 (pMP90) using the freeze–thaw method [45]. Then, *Agrobacterium*-mediated *Arabidopsis* transformation was performed using the floral-dipping method [46]. Transgenic plants were selected on MS plates containing 50 mg/L kanamycin. Homozygous plants of the T_3_ or T_4_ generation were used in this study.

### 4.4. Stress Treatments

For hypoxia treatment, prior to RT-PCR analysis, 10-day-old WT seedlings on MS plates were transferred to filter paper saturated with MS medium and treated with 99.99% nitrogen gas under dark conditions for 0, 1, 2, 4, and 8 h. To analyze phenotypic responses to the hypoxia treatment, 30 seeds of WT plants, *HRE1α* OXs, and *HRE1β* OXs were germinated on the same MS plates. After 7 days, the plates were placed under 99.99% nitrogen gas in the dark for 24 h and then allowed to recover for 2 weeks.

### 4.5. Root Length Measurement

The WT plants, *HRE1α* OXs, and *HRE1β* OXs were germinated on the same MS plates. After 7 days, the plants were pulled out from the MS plates and their primary root lengths were measured.

### 4.6. Yeast Transformation

To investigate transactivation activities in yeast, GAL4 BD-fusion constructs were transformed into a yeast strain, YD116, which harbored *GAL1*::*URA3* and *UAS_GAL4_*::*lacZ* as reporter genes. The yeast transformation was carried out using Frozen-EZ Yeast Transformation II™ kit (Zymo Research Corp., Irvine, CA, USA), according to the manufacturer’s instructions. Transformants were selected on synthetic minimal media lacking tryptophan (SM-Trp).

### 4.7. Transactivation Activity Analysis in Yeast

For yeast growth assay, transformants selected on SM-Trp were streaked onto SM lacking tryptophan and uracil (SM-Trp/-Ura) and incubated at 30 °C for 3–5 days.

Quantitative β-galactosidase assay using ONPG as a substrate was performed according to the methods of Miller et al. [47]. The unit of β-galactosidase activity was then calculated using the formula 1000 × OD_420_/(OD_600_ × assay time in min × assay volume in mL).

For β-galactosidase filter assay, the transformants on SM-Trp were analyzed using 5-bromo-4-chloro-3-indolyl-β-d-galactopyranoside as a substrate. The β-galactosidase filter assay was performed according to the Clontech Yeast Protocols Handbook (Clontech Laboratories, Inc., Mountain View, CA, USA). Reactions were carried out for 6 h.

### 4.8. Protoplast Transformation

*Arabidopsis* protoplast isolation and polyethylene glycol-mediated transformation was performed according to the method described by Yoo et al. [48]. The effector plasmid and reporter plasmid were transformed into *Arabidopsis* protoplasts. A reference plasmid was also cotransformed with these for the normalization of the transformation efficiency. In the transactivation activity analysis, GAL4 BD-fusion constructs under the control of CaMV 35S promoter and firefly luciferase gene under the control of 4× UAS_GAL4_ were used for the effector plasmid and reporter plasmid, respectively. Full-length ORFs of *HRE1α* and *HRE1β* under the control of CaMV 35S promoter and firefly luciferase ORF under the control of 4× GCC box were used for the effector plasmid and reporter plasmid, respectively. Nano luciferase gene under the control of CaMV 35S promoter was used for the reference plasmid.

### 4.9. Dual-Luciferase Assay

Luciferase activity was quantified using the Nano-Glo^®^ Dual-Luciferase^®^ Reporter Assay System (Promega Corp., Madison, WI, USA) and GloMax^®^-Multi Detection System (Promega Corp., Madison, WI, USA), according to the manufacturer’s instructions.

### 4.10. RNA Isolation, cDNA Synthesis, Semi-Quantitative RT-PCR, and Quantitative RT-PCR

Total RNA was isolated using an RNAqueous Kit (Invitrogen, Carlsbad, CA, USA) with Plant RNA Isolation Aid (Invitrogen, Carlsbad, CA, USA), according to the manufacturer’s instructions. Next, 2 μg of total RNA was reverse-transcribed in a total reaction volume of 25 μL; the reaction mixture contained 0.5 μg of oligo-dT primer, 0.5 mM dNTP, 5 μL of 5× reaction buffer, and 200 U of Moloney murine leukemia virus reverse transcriptase (Promega Corp., Madison, WI, USA).

Quantitative RT-PCR was performed in reaction volume of 20 μL; the reaction mixture contained 0.4 μL of cDNA, 10 μL of 2× Power SYBR Green PCR Master mix (Applied Biosystems, Foster, CA, USA), and 0.25 μM gene-specific primers using QuantStudio™ 3 real-time PCR system (Applied Biosystems, Foster, CA, USA). QuantStudio™ Design and Analysis software v.1.4.3 (Applied Biosystems, Foster, CA, USA) was used for the analysis of real-time DNA amplification. The amounts of the target genes expressed were normalized to the expression levels of *GAPc*. The PCR was performed as previously described [49] and all primers used are presented in Table S1.

Semi-quantitative RT-PCR was performed in a reaction volume of 50 μL; the reaction mixture contained 1 μL of cDNA, 0.5 μM gene-specific primers, 0.5 mM of dNTP, 1 U of F-taq DNA polymerase (Solgent, Daejeon, Korea), and 5 μL of 10× reaction buffer. PCR was carried out in 30 cycles for *HRE1α* and *HRE1β* and in 23 cycles for *GAPc*. The number of PCR cycles chosen was in the linear range of the amplification reaction. *GAPc* was amplified as an internal control for the normalization of target gene expression levels. The reaction consisted of an initial denaturation step at 94 °C for 5 min, followed by repeated cycles at 94 °C for 45 s, 56 °C for 45 s, and 72 °C for 45 s, and a final step at 72 °C for 10 min. The primers used in the PCR are provided in Table S1.

### 4.11. Multiple Alignment Analysis

The conserved amino acid sequences in HRE1β and its orthologs were aligned using Clustal Omega (https://www.ebi.ac.uk/Tools/msa/clustalo) and then manually corrected.

### 4.12. Phylogenetic Tree

A phylogenetic tree was generated using Maximum Likelihood method in MEGA 7.0.26 software. The number on each node indicates the bootstrap value for 1000 replicates.

### 4.13. Library Preparation and Sequencing

RNA-Seq was carried out on samples from 10-day-old WT, *HRE1α* OX, and *HRE1β* OX whole seedlings. For control and test RNAs, libraries were constructed using QuantSeq 3′ mRNA-Seq Library Prep Kit (Lexogen, Inc., Vienna, Austria), according to the manufacturer’s instructions. In brief, 500 ng of each total RNA was prepared. Then, an oligo-dT primer containing an Illumina-compatible sequence at its 5′ end was hybridized to the RNA, and reverse transcription was performed. After degradation of the RNA template, second-strand synthesis was initiated by a random primer containing an Illumina-compatible linker sequence at its 5′ end. The double-stranded library was purified using magnetic beads to remove all reaction components. The library was amplified to add the complete adapter sequences required for cluster generation. The finished library was then purified to remove all lingering PCR components. High-throughput sequencing (single-end 75 bp sequencing) was performed with NextSeq 500 (Illumina, Inc., San Diego, CA, USA).

### 4.14. Data Analysis

QuantSeq 3′ mRNA-Seq reads were aligned using Bowtie2 [50]. Bowtie2 indices were either generated from the genome assembly sequences or the representative transcript sequences for the alignment of the genome and transcriptome. The alignment file was used for assembling transcripts, estimating their abundances, and detecting the differential expression of genes. DEGs were determined based on counts from unique and multiple alignments using coverage in Bedtools [51]. The read count data were processed based on the quantile normalization method using EdgeR within R [52] and using Bioconductor [53]. Statistical analyses were performed using two samples *t*-test (equal distribution). The complete mRNA-Seq data from this publication were submitted to the Gene Expression Omnibus database (http://www.ncbi.nlm.nih.gov/geo/) under accession number GSE152166. GO annotation enrichment was performed using DAVID (http://david.abcc.ncifcrf.gov/) with default parameters. Expression analysis in Genevestigator [54] was performed based on the Affymetrix *Arabidopsis* ATH1 Genome Array platform using the condition search tool “perturbations”.

## Figures and Tables

**Table 1:** List of GO terms of genes upregulated more than 2-fold in *HRE1α* OXs.

GO Term	Description	Number in Input List	*p*-Value
GO:0048511	Rhythmic process	5	1.14 × 10^−3^
GO:0009909	Regulation of flower development	5	1.85 × 10^−3^
GO:2000241	Regulation of reproductive process	6	1.90 × 10^−3^
GO:0009628	Response to abiotic stimulus	16	2.54 × 10^−3^
GO:0009416	Response to light stimulus	9	3.08 × 10^−3^
GO:0009314	Response to radiation	9	3.98 × 10^−3^
GO:0048831	Regulation of shoot system development	5	4.85 × 10^−3^
GO:0009639	Response to red or far red light	5	6.66 × 10^−3^
GO:0007623	Circadian rhythm	4	7.67 × 10^−3^
GO:0009719	Response to endogenous stimulus	13	1.46 × 10^−2^
GO:0009739	Response to gibberellin	4	1.49 × 10^−2^
GO:0010468	Regulation of gene expression	18	1.73 × 10^−2^
GO:0048580	Regulation of postembryonic development	5	2.43 × 10^−2^
GO:0048367	Shoot system development	8	2.94 × 10^−2^
GO:0010017	Red or far-red light signaling pathway	3	3.13 × 10^−2^
GO:0051171	Regulation of nitrogen compound metabolic process	17	3.21 × 10^−2^
GO:0071489	Cellular response to red or far red light	3	3.27 × 10^−2^
GO:0010605	Negative regulation of macromolecule metabolic process	6	3.60 × 10^−2^
GO:1901700	Response to oxygen-containing compound	11	3.64 × 10^−2^
GO:0010033	Response to organic substance	13	4.12 × 10^−2^
GO:0019438	Aromatic compound biosynthetic process	18	4.27 × 10^−2^
GO:0033993	Response to lipid	7	4.52 × 10^−2^
GO:0009892	Negative regulation of metabolic process	6	4.75 × 10^−2^
GO:2000112	Regulation of cellular macromolecule biosynthetic process	16	4.86 × 10^−2^
GO:0010556	Regulation of macromolecule biosynthetic process	16	4.99 × 10^−2^

**Table 2:** List of GO terms of genes upregulated more than 2-fold in *HRE1β* OXs.

GO Term	Description	Number in Input List	*p*-Value
GO:0009636	Response to toxic substance	17	2.52 × 10^−11^
GO:1901700	Response to oxygen-containing compound	60	8.75 × 10^−9^
GO:0019748	Secondary metabolic process	29	2.76 × 10^−7^
GO:0009404	Toxin metabolic process	10	6.78 × 10^−7^
GO:0009407	Toxin catabolic process	9	1.16 × 10^−6^
GO:0090487	Secondary metabolite catabolic process	9	1.16 × 10^−6^
GO:0001101	Response to acid chemical	43	6.89 × 10^−6^
GO:0051707	Response to another organism	44	9.27 × 10^−6^
GO:0043207	Response to external biotic stimulus	44	9.59 × 10^−6^
GO:0009607	Response to biotic stimulus	45	1.29 × 10^−5^
GO:0009605	Response to external stimulus	53	1.49 × 10^−5^
GO:0006749	Glutathione metabolic process	9	2.63 × 10^−5^
GO:0034614	Cellular response to reactive oxygen species	8	2.78 × 10^−5^
GO:0071555	Cell wall organization	25	4.92 × 10^−5^
GO:1901698	Response to nitrogen compound	17	5.02 × 10^−5^
GO:0006952	Defense response	50	8.59 × 10^−5^
GO:0045229	External encapsulating structure organization	25	1.29 × 10^−4^
GO:0002213	Defense response to insect	5	2.30 × 10^−4^
GO:0071554	Cell wall organization or biogenesis	28	2.71 × 10^−4^
GO:0010039	Response to iron ion	7	3.12 × 10^−4^
GO:0071732	Cellular response to nitric oxide	5	3.55 × 10^−4^
GO:0006790	Sulfur compound metabolic process	19	3.91 × 10^−4^
GO:0010033	Response to organic substance	57	3.93 × 10^−4^
GO:0006575	Cellular modified amino acid metabolic process	10	3.94 × 10^−4^
GO:0010035	Response to inorganic substance	32	4.05 × 10^−4^

**Table 3:** List of GO terms of genes downregulated more than 2-fold in *HRE1α* OXs.

GO Term	Description	Number in Input List	*p*-Value
GO:0010033	Response to organic substance	14	1.57 × 10^−4^
GO:0009719	Response to endogenous stimulus	13	1.61 × 10^−4^
GO:1901700	Response to oxygen-containing compound	12	2.36 × 10^−4^
GO:0009725	Response to hormone	11	1.59 × 10^−3^
GO:0001101	Response to acid chemical	9	2.49 × 10^−3^
GO:0014070	Response to organic cyclic compound	5	7.19 × 10^−3^
GO:0009751	Response to salicylic acid	4	9.32 × 10^−3^
GO:0009753	Response to jasmonic acid	4	9.81 × 10^−3^
GO:0006355	Regulation of transcription, DNA-template	12	1.29 × 10^−2^
GO:2001141	Regulation of RNA biosynthetic process	12	1.30 × 10^−2^
GO:1903506	Regulation of nucleic acid-templated transcription	12	1.30 × 10^−2^
GO:0051252	Regulation of RNA metabolic process	12	1.46 × 10^−2^
GO:0071229	Cellular response to acid chemical	5	1.61 × 10^−2^
GO:0019219	Regulation of nucleobase-containing compound metabolic process	12	1.70 × 10^−2^
GO:0043161	Proteasome-mediated ubiquitin-dependent protein catabolic process	5	1.90 × 10^−2^
GO:0097659	Nucleic acid-templated transcription	12	1.92 × 10^−2^
GO:0010498	Proteasomal protein catabolic process	5	1.93 × 10^−2^
GO:0032774	RNA biosynthetic process	12	1.95 × 10^−2^
GO:0071310	Cellular response to organic substance	7	1.96 × 10^−2^
GO:0033993	Response to lipid	6	2.05 × 10^−2^
GO:1901698	Response to nitrogen compound	4	2.22 × 10^−2^
GO:2000112	Regulation of cellular macromolecule biosynthetic process	12	2.57 × 10^−2^
GO:0010556	Regulation of macromolecule biosynthetic process	12	2.63 × 10^−2^
GO:0051171	Regulation of nitrogen compound metabolic process	12	3.19 × 10^−2^
GO:0032870	Cellular response to hormone stimulus	6	3.36 × 10^−2^

**Table 4:** List of GO terms of genes downregulated more than 2-fold in *HRE1β* OXs.

GO Term	Description	Number in Input List	*p*-Value
GO:0009628	Response to abiotic stimulus	37	3.49 × 10^−5^
GO:0009719	Response to endogenous stimulus	33	8.07 × 10^−5^
GO:0010033	Response to organic substance	35	2.57 × 10^−4^
GO:0009725	Response to hormone	30	3.11 × 10^−4^
GO:0009061	Anaerobic respiration	4	3.65 × 10^−4^
GO:1901700	Response to oxygen-containing compound	27	1.42 × 10^−3^
GO:0009664	Plant-type cell wall organization	7	2.97 × 10^−3^
GO:0001101	Response to acid chemical	21	4.14 × 10^−3^
GO:0006979	Response to oxidative stress	12	4.59 × 10^−3^
GO:0032870	Cellular response to hormone stimulus	17	5.09 × 10^−3^
GO:0009416	Response to light stimulus	15	5.70 × 10^−3^
GO:0071495	Cellular response to endogenous stimulus	17	6.31 × 10^−3^
GO:0000160	Phosphorelay signal transduction system	8	6.31 × 10^−3^
GO:0009314	Response to radiation	15	8.13 × 10^−3^
GO:0009741	Response to brassinosteroid	5	8.15 × 10^−3^
GO:0042744	Hydrogen peroxide catabolic process	5	8.48 × 10^−3^
GO:0071669	Plant-type cell wall organization or biogenesis	8	9.75 × 10^−3^
GO:0071369	Cellular response to ethylene stimulus	7	1.16 × 10^−2^
GO:0071555	Cell wall organization	12	1.21 × 10^−2^
GO:0010411	Xyloglucan metabolic process	4	1.33 × 10^−2^
GO:0072593	Reactive oxygen species metabolic process	6	1.34 × 10^−2^
GO:0042743	Hydrogen peroxide metabolic process	5	1.35 × 10^−2^
GO:0031328	Positive regulation of cellular biosynthetic process	10	1.38 × 10^−2^
GO:0009755	Hormone-mediated signaling pathway	15	1.42 × 10^−2^
GO:0009891	Positive regulation of biosynthetic process	10	1.58 × 10^−2^

## Supplementary Materials

Supplementary Materials can be found at https://www.mdpi.com/1422-0067/21/19/6984/s1.

## Abbreviations

AP2/ERF	APETALA2/ethylene-responsive factor
BD	DNA-binding domain
CaMV	Cauliflower mosaic virus
DAG	Days after germination
DEG	Differentially expressed gene
DREB/CBF	Dehydration responsive element-binding factor/C-repeat-binding factor
*GAPc*	*Glyceraldehyde 3-phosphate dehydrogenase*
GO	Gene ontology
MS	Murashige and Skoog
ONPG	2-nitrophenyl-β-D-galacto-pyranoside
RAV	Related to ABI3/VP1
