# Supplementary material for: Two Alternative Splicing Variants of AtERF73/HRE1, HRE1α and HRE1β, Have Differential Transactivation Activities in Arabidopsis
Source: Int J Mol Sci. 2020 Sep 23;21(19):6984. doi: 10.3390/ijms21196984 (PMC7582492; doi:10.3390/ijms21196984)
Supplement: Supplementary file 1 [file ijms-21-06984-s001.zip › Supplementary data-ijms-934917-revised.docx]

**Supplementary data**

**Table S1. List of primers for RT-PCR**

| **Gene** | **Forward** | **Orientation** | **Purpose** |
| --- | --- | --- | --- |
| *HRE1α* | 5′-CTTTATCCATGGCTAGGCTC-3′ | Forward | Quantitative RT-PCR |
| *HRE1α* | 5′-GCCAAACTCGAACTCCTT TG-3′ | Reverse | Quantitative RT-PCR |
| *HRE1β* | 5′-CATGCTTCAGGCTCAGCATC-3′ | Forward | Quantitative RT-PCR |
| *HRE1β* | 5′-GCCAAACTCGAACTCCTTTG-3′ | Reverse | Quantitative RT-PCR |
| *WRKY46* | 5′-CTATTGATGATGGTCACTGC-3′ | Forward | Quantitative RT-PCR |
| *WRKY46* | 5′-TGTGAATCGATGCGTGCATC-3′ | Reverse | Quantitative RT-PCR |
| *BBX16*/*EIP6* | 5′-TTGGTGTGGTAAGTTAGGAC-3′ | Forward | Quantitative RT-PCR |
| *BBX16*/*EIP6* | 5′-CTAAGAACACTCTCTTCGTC-3′ | Reverse | Quantitative RT-PCR |
| At2g46670 | 5′-CGAGAAAGCAGCTAGCAAGG-3′ | Forward | Quantitative RT-PCR |
| At2g46670 | 5′-CGTTGTTTCGCACTTTCTTC-3′ | Reverse | Quantitative RT-PCR |
| *ASG4* | 5′-TCACATCCGGTGATTAGCAC-3′ | Forward | Quantitative RT-PCR |
| *ASG4* | 5′-CAACAGTTCTCTGAGACACC-3′ | Reverse | Quantitative RT-PCR |
| *AGP30* | 5′-AGATGCGGTGGTGAGACTTG-3′ | Forward | Quantitative RT-PCR |
| *AGP30* | 5′-TATCGTAGTTGGTCACCGTC-3′ | Reverse | Quantitative RT-PCR |
| *BBE8* | 5′-TGGGGAGGATATCATCTACG-3′ | Forward | Quantitative RT-PCR |
| *BBE8* | 5′-CATGTACCGGTCGGTTAAAG-3′ | Reverse | Quantitative RT-PCR |
| *GAPc* | 5′-GTGTCCCAACCGTTGATGTC-3′ | Forward | Quantitative RT-PCR |
| *GAPc* | 5′-TCCCTTGAGTTTGCCTTCGG-3′ | Reverse | Quantitative RT-PCR |
| *HRE1α* | 5′-CTTTATCCATGGCTAGGCTC-3′ | Forward | Semi-quantitative RT-PCR |
| *HRE1α* | 5′-GCATCGCCCATGATGATATC-3′ | Reverse | Semi-quantitative RT-PCR |
| *HRE1β* | 5′-CATGCTTCAGGCTCAGCATC-3′ | Forward | Semi-quantitative RT-PCR |
| *HRE1β* | 5′-GCATCGCCCATGATGATATC-3′ | Reverse | Semi-quantitative RT-PCR |
| *GAPc* | 5′-CACTTGAAGGGTGGTGCCAAG-3′ | Forward | Semi-quantitative RT-PCR |
| *GAPc* | 5′-CCTGTTGTCGCCAACGAAGTC-3′ | Reverse | Semi-quantitative RT-PCR |


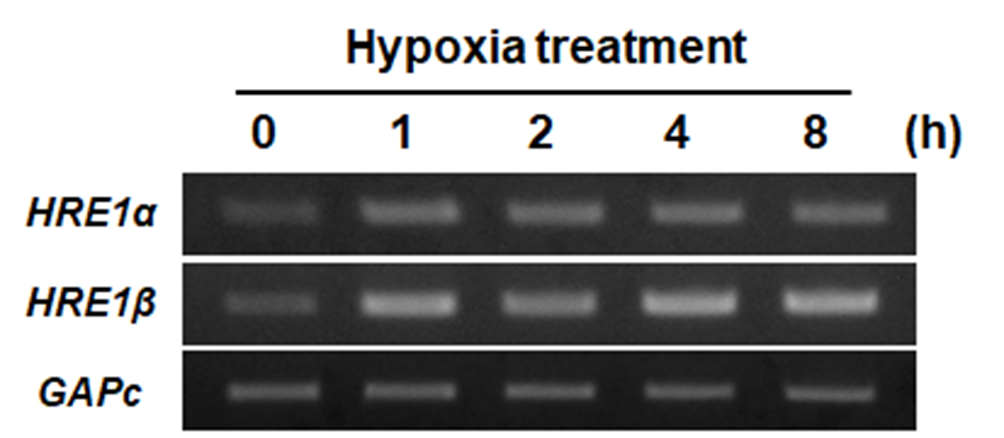


**Figure S1. Expression analysis of *HRE1α* and *HRE1β* under hypoxia condition.** The expression patterns of *HRE1α* and *HRE1β* under hypoxic conditions using semi-quantitative RT-PCR. Hypoxia was treated with 99.99% nitrogen gas for 0, 1, 2, 4, and 8 h. *GAPc* was used as an internal control. Similar results were obtained from at least two biological replicates, with one shown here.


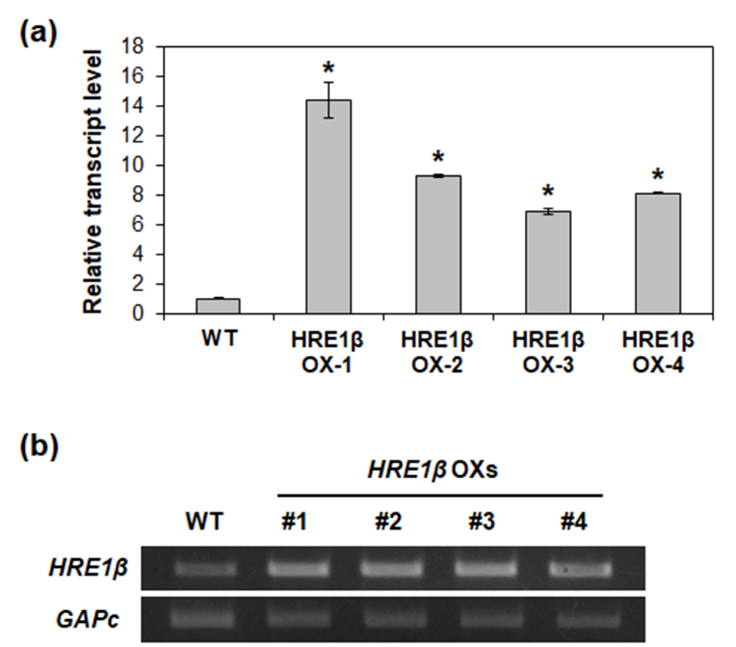


**Figure S2. Generation and selection of *HRE1β* OXs.** (**a**) Selection of the T_1_ lines of *HRE1β*-overexpressing transgenic plants (OXs) by quantitative RT-PCR analysis. *GAPc* was used as an internal control. The transcript level in WT was set as 1. Three independent reactions were performed for each technical replicate. Two technical replicates were performed for each biological replicate. At least two biological replicates showed similar results, with one shown here. The data shown are the means ± S.D. (*n* = 6). * indicates *t*-test *P* < 0.05. (**b**) Selection of the T_1_ lines of *HRE1β* OXs by semi-quantitative RT-PCR analysis. *GAPc* was used as an internal control. Similar results were obtained from at least two biological replicates, with one shown here.


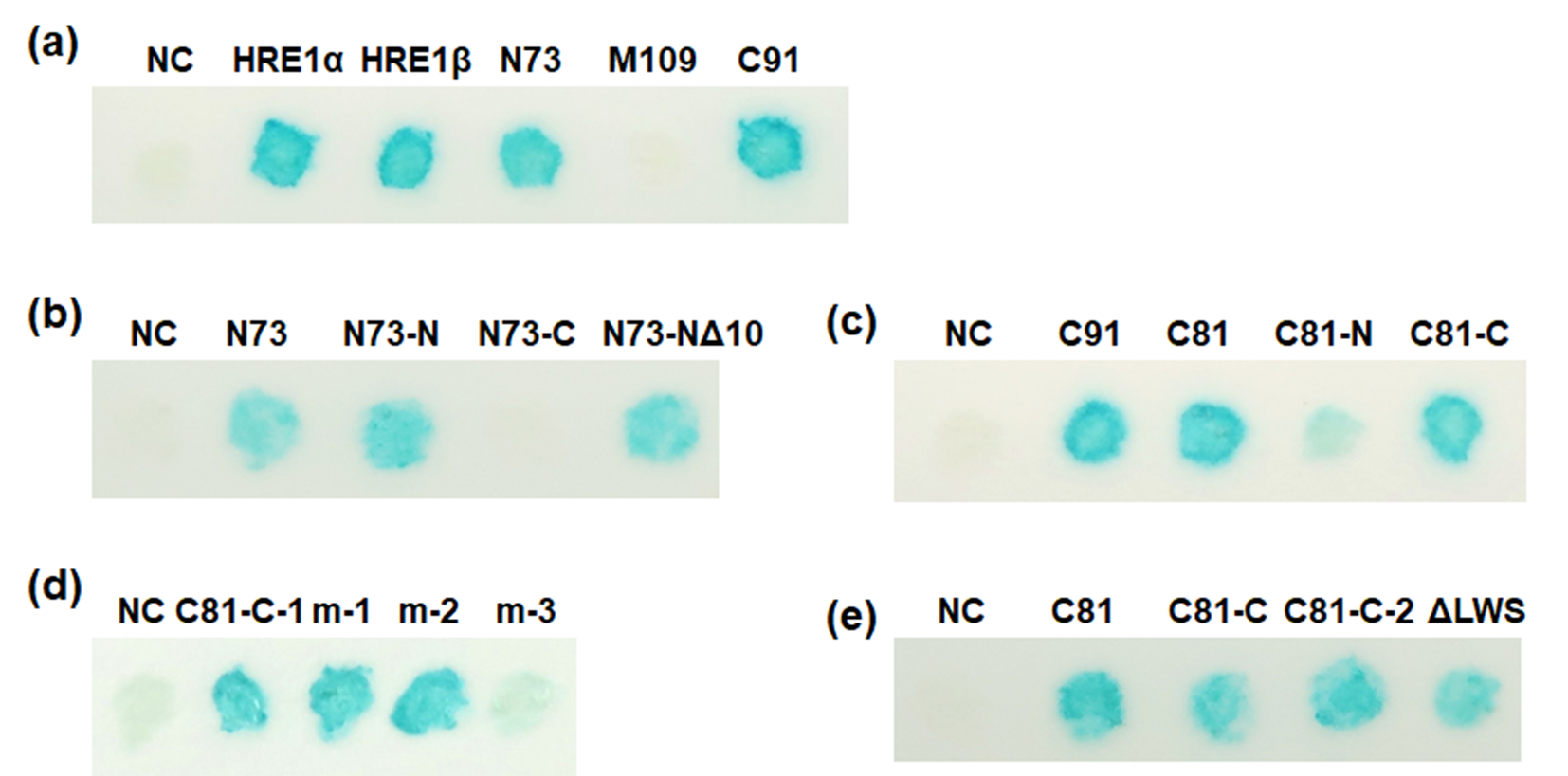


**Figure S3. Analysis of transactivation activities of HRE1α and HRE1β.** Transactivation activities of full-length ORFs of HRE1α and HRE1β, and truncated fragments of HRE1β using β-galactosidase filter assay. 5-bromo-4-chloro-3-indolyl-β-d-galactopyranoside was used as a substrate. The reaction was performed for 6 h.

**Table S2. Suitability of RNA-Sequencing data**

| **Sample** | **Total_read** | **Processed_read** | **Mapped_read** | **Mapping_rate** |
| --- | --- | --- | --- | --- |
| WT-1 | 31,584,235 | 30,119,902 | 29,519,240 | 98.01% |
| WT-2 | 32,020,154 | 30,339,538 | 29,616,323 | 97.62% |
| HRE1α OX-1 | 29,393,426 | 27,783,397 | 26,765,028 | 96.33% |
| HRE1α OX-2 | 31,577,495 | 29,912,243 | 28,874,530 | 96.53% |
| HRE1β OX-1 | 28,834,110 | 27,353,383 | 26,698,926 | 97.61% |
| HRE1β OX-2 | 33,917,182 | 31,958,661 | 31,182,230 | 97.57% |


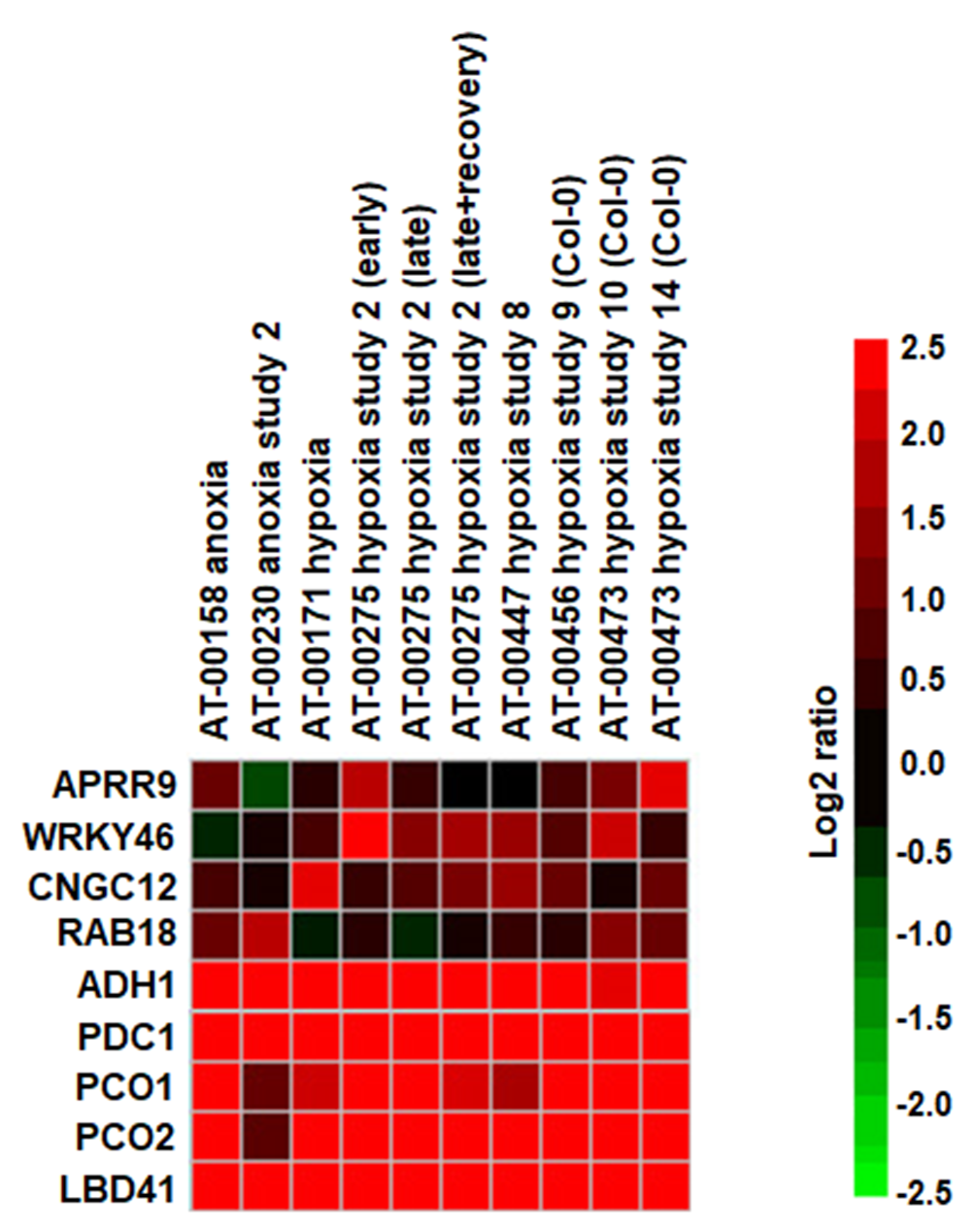


**Figure S4. Heatmap analysis of more than 2-fold upregulated genes in *HRE1α* OXs under hypoxia conditions using Genevestigator.** *ADH1*, *PCD1*, *PCO1*, *PCO2*, and *LBD41* were used as marker genes for hypoxic stress condition.


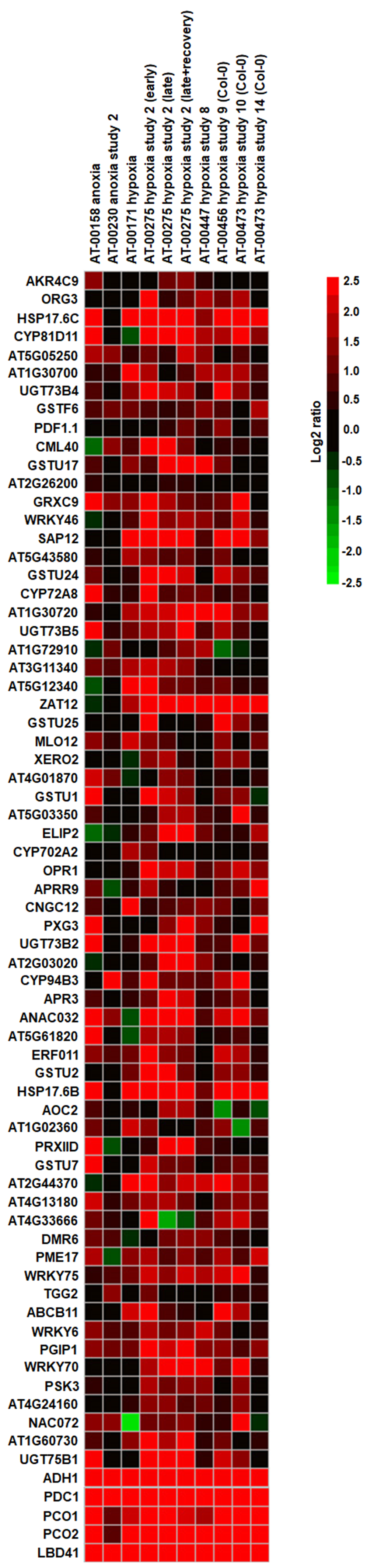


**Figure S5. Heatmap analysis of more than 2-fold upregulated genes in *HRE1β* OXs under hypoxia conditions using Genevestigator.** *ADH1*, *PCD1*, *PCO1*, *PCO2*, and *LBD41* were used as marker genes for hypoxic stress condition.

**Table S5. List of primers for cloning**

| **Construct** | **Sequence** | **Orientation** |
| --- | --- | --- |
| HRE1β entire ORF | 5′-CGCGAATTCATGTGCGGAGGAGCTGTAAT-3′ | Forward |
| HRE1β entire ORF | 5′-CAGGTCGACTCAGGACCATAGACCCATGT-3′ | Reverse |
| HRE1β M109 | 5′-GTCGAATTCGGCTCAGCATCAGATGGGAA-3′ | Forward |
| HRE1β M109 | 5′-ACAGTCGACAAGACAGCTACTACTAGGCG-3′ | Reverse |
| HRE1β N73 | 5′-CGCGAATTCATGTGCGGAGGAGCTGTAAT-3′ | Forward |
| HRE1β N73 | 5′-CACGTCGACTCAGCTCTGTTTCTTCTTCCCAT-3′ | Reverse |
| HRE1β N73-N | 5′-CGCGAATTCATGTGCGGAGGAGCTGTAAT-3′ | Forward |
| HRE1β N73-N | 5′-CGCGTCGACTCAGGACTCTAATTCATCGAAAT-3′ | Reverse |
| HRE1β N73-C | 5′-CGCGAATTCGATGAGCCATTTGTCTTCTC-3′ | Forward |
| HRE1β N73-C | 5′-CACGTCGACTCAGCTCTGTTTCTTCTTCCCAT-3′ | Reverse |
| HRE1β N73-NΔ10 | 5′-CGCGAATTCATAGCGCCGGAGAAGATTGC-3′ | Forward |
| HRE1β N73-NΔ10 | 5′-CGCGTCGACTCAGGACTCTAATTCATCGAAAT-3′ | Reverse |
| HRE1β C81 | 5′-CGCGAATTCACGCTTCTGATTGATACACA-3′ | Forward |
| HRE1β C81 | 5′-CAGGTCGACTCAGGACCATAGACCCATGT-3′ | Reverse |
| HRE1β C81-N | 5′-CGCGAATTCACGCTTCTGATTGATACACA-3′ | Forward |
| HRE1β C81-N | 5′-CGCGTCGACTCAGTTGGCTTCTTCACTATCAT-3′ | Reverse |
| HRE1β C81-C | 5′-CGCGAATTCAACGTTGATGCTTCTCTGCT-3′ | Forward |
| HRE1β C81-C | 5′-CAGGTCGACTCAGGACCATAGACCCATGT-3′ | Reverse |
| HRE1β C81-C-1 | 5′-CGCGAATTCAACGTTGATGCTTCTCTGCT-3′ | Forward |
| HRE1β C81-C-1 | 5′-ACAGTCGACTCACGTAAAAGGCATCTGCGAGA-3′ | Reverse |
| HRE1β C81-C-2 | 5′-CGCGAATTCGAGGGAAACTGTGATTCCTC-3′ | Forward |
| HRE1β C81-C-2 | 5′-CAGGTCGACTCAGGACCATAGACCCATGT-3′ | Reverse |
| HRE1β C81-C-2ΔLWS | 5′-CGCGAATTCGAGGGAAACTGTGATTCCTC-3′ | Forward |
| HRE1β C81-C-2ΔLWS | 5′-CAGGTCGACTCAGTCATTGCCTCCATCAAAGA-3′ | Reverse |
| HRE1α entire ORF | 5′-CGCTCTAGAATGTCTCAAAGCTTTGAACT-3′ | Forward |
| HRE1α entire ORF | 5′-ATACCCGGGTCAGGACCATAGACCCATGT-3′ | Reverse |
| HRE1β entire ORF | 5′-CGCTCTAGAATGTGCGGAGGAGCTGTAAT-3′ | Forward |
| HRE1β entire ORF | 5′-ATACCCGGGTCAGGACCATAGACCCATGT-3′ | Reverse |
| HRE1β N73 | 5′-CGCTCTAGAATGTGCGGAGGAGCTGTAAT-3′ | Forward |
| HRE1β N73 | 5′-ATACCCGGGTCAGCTCTGTTTCTTCTTCCCAT-3′ | Reverse |
| HRE1β M109 | 5′-CGCTCTAGAGGCTCAGCATCAGATGGGAA-3′ | Forward |
| HRE1β M109 | 5′-ATACCCGGGTCAAAGACAGCTACTACTAGGCG-3′ | Reverse |
| HRE1β C81 | 5′-CGCTCTAGAACGCTTCTGATTGATACACA-3′ | Forward |
| HRE1β C81 | 5′-ATACCCGGGTCAGGACCATAGACCCATGT-3′ | Reverse |
